# Sleep Spindles as an Electrographic Element: Description and Automatic Detection Methods

**DOI:** 10.1155/2016/6783812

**Published:** 2016-07-11

**Authors:** Dorothée Coppieters 't Wallant, Pierre Maquet, Christophe Phillips

**Affiliations:** ^1^GIGA In Silico Medicine, CRC (B30), University of Liège, 4000 Liège, Belgium; ^2^Department of Electrical Engineering and Computer Science (B28), 4000 Liège, Belgium; ^3^GIGA CRC In Vivo Imaging, CRC (B30), University of Liège, 4000 Liège, Belgium; ^4^Department of Neurology, University of Liège Hospital (B35), 4000 Liège, Belgium; ^5^Walloon Excellence in Life Sciences and Biotechnology (WELBIO), 1300 Wavre, Belgium

## Abstract

Sleep spindle is a peculiar oscillatory brain pattern which has been associated with a number of sleep (isolation from exteroceptive stimuli, memory consolidation) and individual characteristics (intellectual quotient). Oddly enough, the definition of a spindle is both incomplete and restrictive. In consequence, there is no consensus about how to detect spindles. Visual scoring is cumbersome and user dependent. To analyze spindle activity in a more robust way, automatic sleep spindle detection methods are essential. Various algorithms were developed, depending on individual research interest, which hampers direct comparisons and meta-analyses. In this review, sleep spindle is first defined physically and topographically. From this general description, we tentatively extract the main characteristics to be detected and analyzed. A nonexhaustive list of automatic spindle detection methods is provided along with a description of their main processing principles. Finally, we propose a technique to assess the detection methods in a robust and comparable way.

## 1. Introduction

Spindles are intriguing brain oscillations observed in particular stages of sleep (NREM sleep) and during anesthesia. They constitute the first characteristic sleep patterns observed in electroencephalographic (EEG) signal whose neurophysiological mechanisms were elucidated [1, 2]. The first hint for the involvement of thalamocortical loops in their generation dates back to 1945 [3, 4]. The first mechanistic models were proposed in the 90s [5–8] and were regularly updated since then [9–13]. According to these models, spindle generation mainly depends on an interplay between thalamus and cortex, including the hippocampus [12]. Because thalamocortical loops play a key role in buttressing brain function, it is not surprising that modifications of spindle characteristics were reported in a wide array of physiological and pathological conditions: learning and memory [13–15], aging [16, 17], and neurodegenerative and psychiatric diseases [18]. Likewise, the interindividual variability of spindles is large [19] compared to their intraindividual stability [20] or to their similarity between monozygotic twins [21]. They also appear under various species-specific blends in rodents [9], carnivores [22, 23], nonhuman primates [24], and humans.

Despite the wealth of data accumulated about these oscillations, spindles continue to raise a number of questions. The mechanisms underpinning the functional impact, sometimes referred to as function, of spindles remain obscure and were the focus of several hypotheses [23, 25]. Even the development of a generic, systematic, reproducible, and robust method to detect and analyze sleep spindles proved surprisingly difficult [26].

This review aims to capture the various aspects of spindles that should be accounted for by such “automatic spindle detection methods” (ASDM). The review consists of two main parts. The first part describes the sleep spindle physically (Section 2.1) and topographically (Section 2.2). The second part describes the general workflow of ASDM (Section 3) as well as each part of it (Sections 3.4 and 3.3) and then proposes a consensus method to assess ASDM performance (Section 4). Finally, a discussion and conclusion are given (Section 5).

## 2. Spindle Description

In 1968, Rechtschaffen and Kales defined spindles as “…* a burst of oscillatory brain activity visible on an EEG that occurs during stage 2 sleep. It consists of 12–14 Hz waves that occur for at least 0.5 seconds*” [27]. More recently, Iber et al. specified in the manual of the American Academy of Sleep Medicine [28] the following: “*Sleep spindle [is] a train of distinct waves with frequency 11–16 Hz (most commonly 12–14 Hz) with a duration ≥0.5 seconds, usually maximal in amplitude using central derivations*.” Still there is some freedom in how spindles are described individually (Section 2.1) but also in terms of their variability (Section 2.2).

### 2.1. Physical Description

The physical description defines the spectral (frequency and intrafrequency) and temporal (shape and duration) properties of the sleep spindle.

#### 2.1.1. Main Frequency

No neurophysiological argument can still delimit definitely the frequency range of a sleep spindle. Indeed, spindle frequency variability has been identified between individuals (twins [21], age [16, 17]), within individuals (across the night [20]), and even between spindles [29]. In animals, spindles frequency can range from 7–15 Hz in cats [22] to 6–10 Hz in ferrets [9]. In humans, the spindle frequency range is typically between 11 and 16 Hz, although some authors report much slower (9-10 Hz [30, 31]) and faster (18 Hz [32]) spindles. Furthermore, (at least) two types of spindles are reported in humans: late slow frontal (9–12 Hz) and early fast centroparietal (13–15 Hz) spindles (reviewed in [18, 33]). This dichotomy was reported in various EEG and MEG studies [13, 18, 20, 30] but also with functional magnetic resonance imaging (fMRI) [34] and after pharmacological manipulation [35]. This distinction, based on the spindle time onset, frequency, and topography, also corresponds to different phase relationships with sleep slow oscillations and implications in memory consolidation [12, 14, 15, 31] (Section 2.2).

#### 2.1.2. Intraspindle Frequency

**Figure 1:**
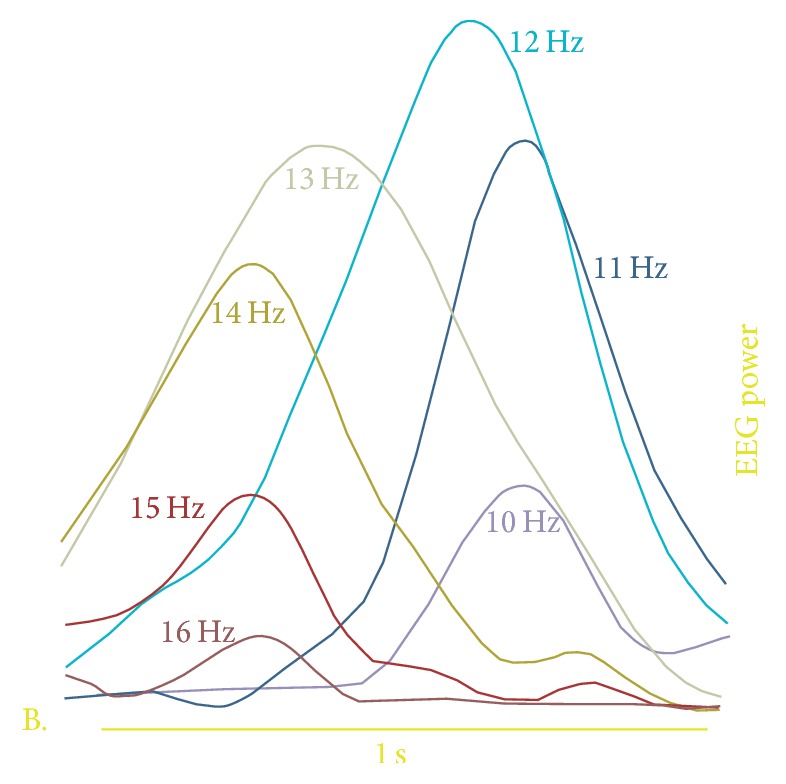
The intraspindle frequency variation. Averaged (60 EEG sensors) spatiotemporal evolution of different frequency during an example spindle. Power in different frequencies is color coded (warmer colors are higher frequencies). Modified from [37].

Changes in intraspindle frequency were recently reported, a phenomenon attributed to variations in levels of depolarization of thalamocortical (TC) neurons [30]. This phenomenon was observed with both EEG and MEG [36–38] but also using deep brain recordings [30, 39]. On average, 50% spindles show a decrease in intraspindle frequency (Figure 1) [30, 36–40]. On average, the oscillation deceleration has been estimated around −0.8 Hz/s over a large population of spindles [30].

#### 2.1.3. Duration

The minimum duration of a spindle has been set to 0.5 s seemingly without any firm objective physiological criterion. Admittedly a minimum number of oscillations are necessary to estimate spindle frequency or its typical waxing waning shape. However, some authors now detect sleep spindles as short as 0.3 s [26].

By contrast, no maximal duration value has been proposed. Yet, spindles are discrete events, a feature that distinguishes them from spindling activity (i.e., the continuous EEG activity between 11 and 16 Hz, often reported as the EEG power in this frequency band). Different neurophysiologic mechanisms were proposed to explain spindle termination [13, 41]: the refractory period due to sustained open calcium channels [9], the intra-spell-out regulation [12], and the desynchronization of cortical neurons [42].

On EEG recordings, the spindle duration critically depends on its delimitation with the identification of spindle start and end which is heavily dependent on frequency analysis specifications (when the signal is filtered in a specific frequency range) and/or on an arbitrary amplitude threshold (Section 3.2).

No direct link has been found between spindle duration and frequency. Nevertheless, both are considered as very stable characteristics from night to night in an individual (reviewed in [26]) whereas they vary substantially between individuals [20].

#### 2.1.4. Waxing Waning Shape

**Figure 2:**
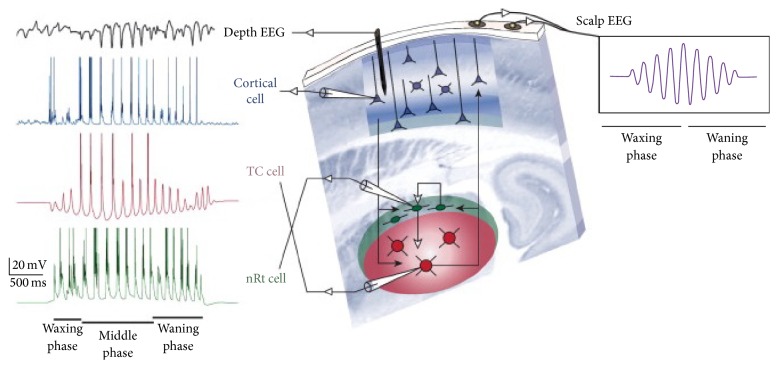
Spindles are generated in thalamocortical (TC) loop. The reticular (nRt) cells encounter the TC cells confined within the thalamus. The nRt cells inhibit TC cells which project excitatory inputs to the cortical cells. Cortical cells send excitatory input back to thalamic neurons. Sleep spindles arise from a cascade of recurrent, inhibitory, and excitatory signals between nRt, TC, and cortical cells. Modified from [18].

Sleep spindles typically have a symmetrical waxing and waning shape [26]. The reasons for this morphological aspect are not fully understood. During the initial part of a spindle, the population of cortical neurons recruited synchronously by the oscillation grows. Consequently, on EEG recordings the amplitude of spindle oscillations increases: it corresponds to the waxing phase (Figure 2). In the second phase, different neuronal mechanisms are thought to induce a reduction or a dephasing of recruited neuronal population leading to the waning phase during which the EEG amplitude of oscillations decreases [41] (Figure 2).

### 2.2. Topographical Description

During a burst of successive sleep spindles (5–15 events) [13, 43], typically recurring every 4 s [16, 42–45], each sleep spindle is described as late slow frontal or early fast centroparietal according to its time onset, its mean frequency, and its position on the median line. Spindle can also be described as local or global according to its visibility over scalp and isolated or combined with a Slow Wave Oscillation. Moreover, according to analysis done simultaneously in EEG and MEG, some sleep spindles are only visible in one modality only. This differentiation gives rise to a new TC model [46] which could explain the presence of focal spindles [30, 39].

#### 2.2.1. Late Slow Frontal versus Early Fast Centroparietal Spindles

**Figure 3:**
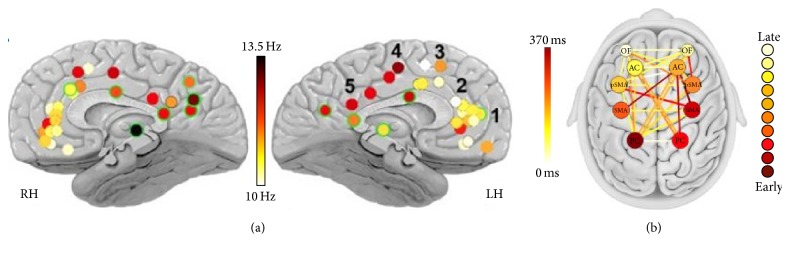
Fast and slow sleep spindles. (a) Average frequency of spindles across depth electrodes (*n* = 50 electrodes in 13 individuals). The color of each circle denotes the mean spindle frequency in an individual electrode according to its precise anatomical location. Green outlines mark electrodes placed more laterally than the midline. The two outliers in the medial prefrontal cortex (red circles) were the only electrode placements in one atypical individual in whom parietal spindles may be even faster than 13.5 Hz. (b) Quantitative analysis of time offsets in spindle occurrence. A graph showing the order in which spindles are detected across multiple regions (node color) and the mean temporal delays within each pair of regions (edge color). Mean order and timing across spindles for all individuals (*n* = 12) indicate that centroparietal spindles precede frontal spindles. Orbitofrontal cortex (OF), anterior cingulate cortex (AC), posterior cingulate cortex (PC), presupplementary motor area (pSMA), and supplementary motor area (MSA). Modified from [30].

This distinction between late slow frontal and early fast centroparietal spindles comes from observations done in EEG and MEG but has also been reinforced with fMRI [34] and pharmacological manipulation [35]. Sleep spindle frequency is related to its location, with a relative decrease of the mean frequency from centroparietal to frontal areas. This difference between faster and slower spindles would be more pronounced in deeper brain areas with a sharp distinction around the supplementary motor area (SMA) (Figure 3(a)) [30]. In [38], Zerouali et al. proposed that the mean frequency observed could be relative to the duration of the fast and slow regimes composing the spindle itself depending on network properties [47, 48]. On average fast centroparietal spindles precede slow frontal spindles by 200–500 ms whereas no significant differences are found within both regions [30, 31, 49] (Figure 3(b)).

#### 2.2.2. EEG versus MEG Spindles

**Figure 4:**
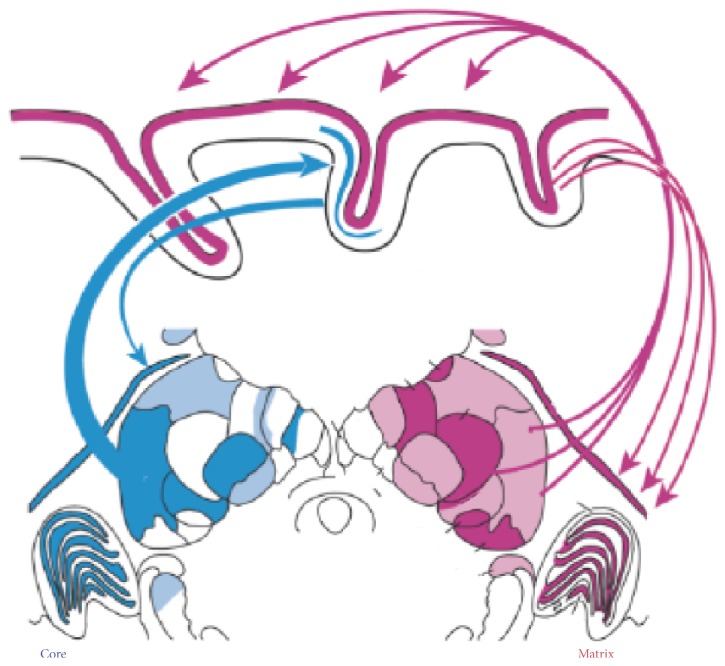
A thalamocortical system to reconcile differences between EEG and MEG sleep spindles. The distribution of matrix cells (red) and core cells (blue) indicated in a frontal section through the middle of a macaque monkey thalamus. The core thalamic projections are topographically ordered to the middle layers of a single cortical field. The matrix thalamic projections collectively project diffusely to the superficial layers of widespread cortical fields. Modified from [24].

It is admitted that spindles recorded in EEG and MEG come from dissociated generators [50–53]. This postulate is due to differences observed between EEG and MEG spindles: the coherence is larger between pairs of EEG than MEG sensors (resp., 0.7 compared to 0.3 [52]); spindles are even sometimes only visible in one of the two modalities [52, 53]. Typically, MEG spindles precede EEG spindles. Bonjean et al. account for this finding by assuming two subpopulations of thalamocortical cells: core and matrix TC cells [42, 46] (Figure 4). The former, the core pathway, projects focally to middle cortical layers in specific cortical areas whereas the latter sends projections diffusely to superficial cortical layers of widespread cortical regions [24, 54, 55]. This model reconciles the difference in MEG and EEG as follows: spindles would first focally occur in the core pathway (MEG spindles) and then quickly spread to the matrix pathway (EEG spindles) which contributes to their widespread synchronization across the cortical network [46]. This hypothesis is supported by observations in humans [52, 53].

In theory, the displacement of electric charges induced by neuronal activity produces both electric and magnetic fields observable, respectively, from EEG and MEG recordings. Therefore, beyond these different source dynamics, differences in scalp topography are also partially explained by the way the electric and magnetic fields project on the scalp: EEG (resp., MEG) sensors show higher sensitivity for sources oriented radially (resp., tangentially) to the scalp surface. Moreover the low-conductivity of the skull induces large spreading, that is, spatial blurring, in the EEG contrary to MEG. Finally in MEG, different sensor types are used, mainly magnetometer and radial/planar gradiometer, each having a specific sensitivity profile.

Still sleep spindle characteristics observed from both modalities should be the same with only differences in terms of spatial distribution [56, 57]. This means that ASDM used in EEG should be transposable to MEG signals.

#### 2.2.3. Local versus Global Spindles

Although spindles are usually analyzed across multiple recording sites (global spindles), some spindles could also appear independently, in separate brain regions (local spindles). The regional aspect of sleep spindles is interpreted as a consequence of the reprocessing by sleep spindles of specific memory traces [58].

**Figure 5:**
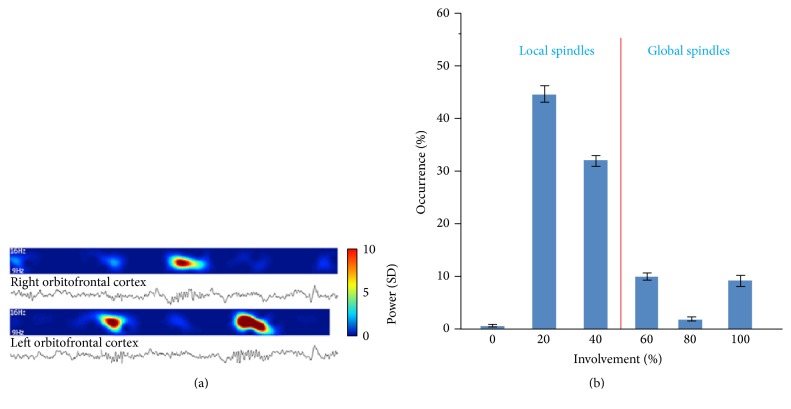
Local spindles. (a) An example of local spindles. Depth EEG along with corresponding spectrograms in the spindle frequency range (9–16 Hz) during 15 s of slow wave sleep. (b) Distribution of involvement (percent of monitored brain structures expressing each spindle). Considering local spindle below 50% of involvement, most sleep spindles are local (21240 spindles in 49 electrodes of 12 individuals). Modified from [30].

This differentiation is particularly reported in deeper brain areas (via MEG [52, 53] or intracerebral electrodes (EEG) [30, 39, 44]) with actually more local spindles than global ones (73% of all spindles are observed in less than half the electrodes [30]) (Figure 5). The comparison of deeper homotopic regions also revealed many spindles observed only in one hemisphere (40.4 ± 1.7% [39]), indicating that differences between anterior and posterior regions could not account for local spindles [39].

The observation of local and global spindles could also be explained by the model proposed by Bonjean et al. [46]: spindles would first come from the core pathway focally (local spindles) and then spread to the matrix pathway more synchronized (global spindles) [30, 39, 46].

The distinction between early fast centroparietal and late slow frontal spindles as well as the local and global spindles should encourage people to analyze and detect spindles over all channels.

#### 2.2.4. Oscillatory Context of Spindles

Sleep spindles are just one type of EEG oscillating waves observed during NREM sleep which is mainly characterized by three rhythms: Slow Wave Oscillation (SWO) (≤1 Hz), delta oscillation (1–4 Hz), and sleep spindles (7–15 Hz) (reviewed in [22]). However, other rhythms are also detected during NREM sleep: cortical gamma rhythms (30–80 Hz), cortical or hippocampal ripples, and neocortical ripples (>100 Hz) (reviewed in [11]).

Thalamic delta oscillations (1–4 Hz) were shown to arise from the interplay between a nonspecific cation current and the low threshold Ca^2+^ current. At the cortical level, they are usually considered as SWO generated under conditions of low sleep pressure [59]. The SWO (≤1 Hz) is a fundamental sleep oscillation characterized, at the cellular level, by a bistable membrane potential that alternates between a hyperpolarized state, during which the neurons are silent, and a depolarized state, during which the neurons fire. During hyperpolarization, the thalamus is disabled and no sustained activity exists between thalamus and cortex whereas during active state the thalamus generates fast oscillations within thalamocortical network. This oscillation seems to have its origin in cortical area [11] but the thalamus contributes to its generation [60].

SWO, sleep spindles, and ripples in hippocampus are hierarchically nested [61, 62]. The nesting of frequencies results in synchrony over widespread regions in brain activity [61, 63–65]: ripples occur during troughs of sleep spindles whereas sleep spindles are mainly observed during troughs of slow oscillations (in deep area) [61].

**Figure 6:**
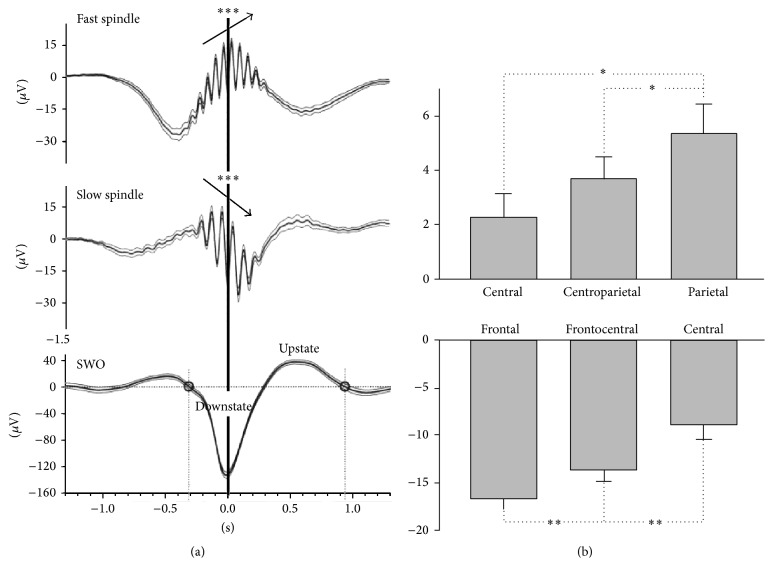
Late slow and early fast spindles with the Slow Wave Oscillation (SWO). (a) Grand mean averages (±SEM) of original EEG in (top) 12 centroparietal channels across all detected fast spindles, in (middle) 12 frontocentral channels across all detected slow spindles, and (bottom) in 8 frontocentral channels across all detected slow oscillations. Note that spindles are averaged independently of whether an SWO was present. Averaging was performed with reference to the deepest, that is, most negative, trough in the filtered signal (*t* = 0). Asterisks (and arrows) indicate a significant (*P* < 0.001) positive and negative slow potential shift underlying early fast and late slow spindles, respectively, in the interval between 300 ms before and 300 ms after the spindle peak. (b) Potential shifts underlying early fast spindles and late slow spindles. Mean (±SEM) slow potential shifts underlying (top) early fast spindles at central, centroparietal, and parietal electrodes and (bottom) late slow spindles at frontal, frontocentral, and central electrodes. Asterisks indicate significant differences between topographies in positive and negative slow potential shifts, respectively (^*∗∗*^ *P* < 0.01, ^*∗*^ *P* < 0.05). Modified from [31].

Significant differences were found in the association of early fast and late slow spindles with SWO. Taking the most negative trough of the SWO as the reference time (*t* = 0), early fast centroparietal spindles appear to ride on the positive (going upward) potential of the SWO whereas late slow frontal spindles appear on the downward side of the SWO [30, 31] (Figure 6(a)). The shift between the spindle peak and the reference time (*t* = 0) is more positive, the more posterior the fast spindle, and more negative, the more anterior the slow spindle (Figure 6(b)). This observation is in keeping with the fact that the SWO is a traveling wave going from the anterior to the posterior brain areas [66].

**Figure 7:**
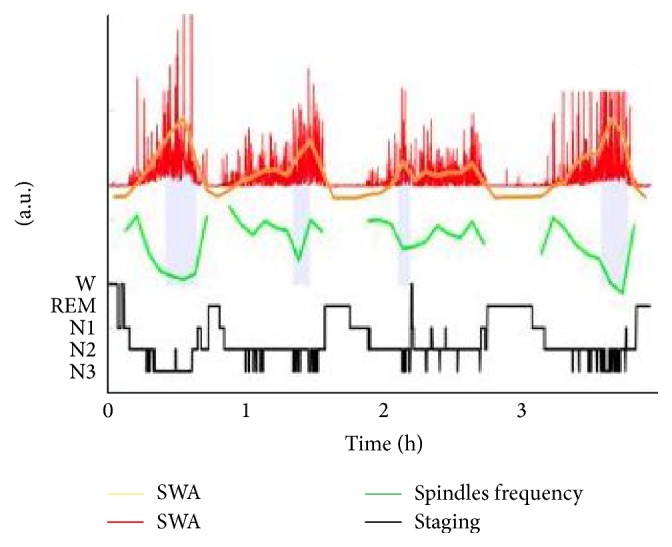
Example of time course of Slow Wave Activity (SWA) and spindle frequency dynamics throughout sleep in the anterior cingulate of one individual. Note that within NREM cycles, spindle frequency is lowest when SWA is highest (vertical purple bars) and increases towards transitions to REM sleep. Modified from [30].

With intracerebral analysis, Nir et al. [39] showed that 53.7% ± 3.1% of sleep spindles were not associated with SWO (no SWO around sleep spindle ±1.5 s) and among these isolated spindles, 79.8% were considered as local spindles. In deeper brain areas, sleep spindles and SWO are not so much associated. However, at the scalp level, the mean spindle frequency is on average slower during sustained SWO (0.5–4 Hz, corresponding to slow wave sleep) than during light sleep (N2) [30, 67]. Deeper sleep is thus associated with lower spindles frequencies and density [30]. Moreover, this differentiation in spindle frequency over sleep stages was observed within NREM sleep. During the first four cycles, the time courses of mean spindles frequency and slow waves activities (SWA) have been associated with a U-shape [68] and an inverse U-shape [30], respectively (Figure 7).

#### 2.2.5. Source Localization

Spindles are observed most often through the TC loop (thalamus, cortex, and neocortex). Nevertheless, with intracerebral EEG, some spindles have also been observed in parahippocampal gyrus, hippocampus, and amygdala [30, 65]. A few spindles have also been observed in medial temporal lobe but this could be due to pathology in epilepsy patients [30, 69, 70].

**Figure 8:**
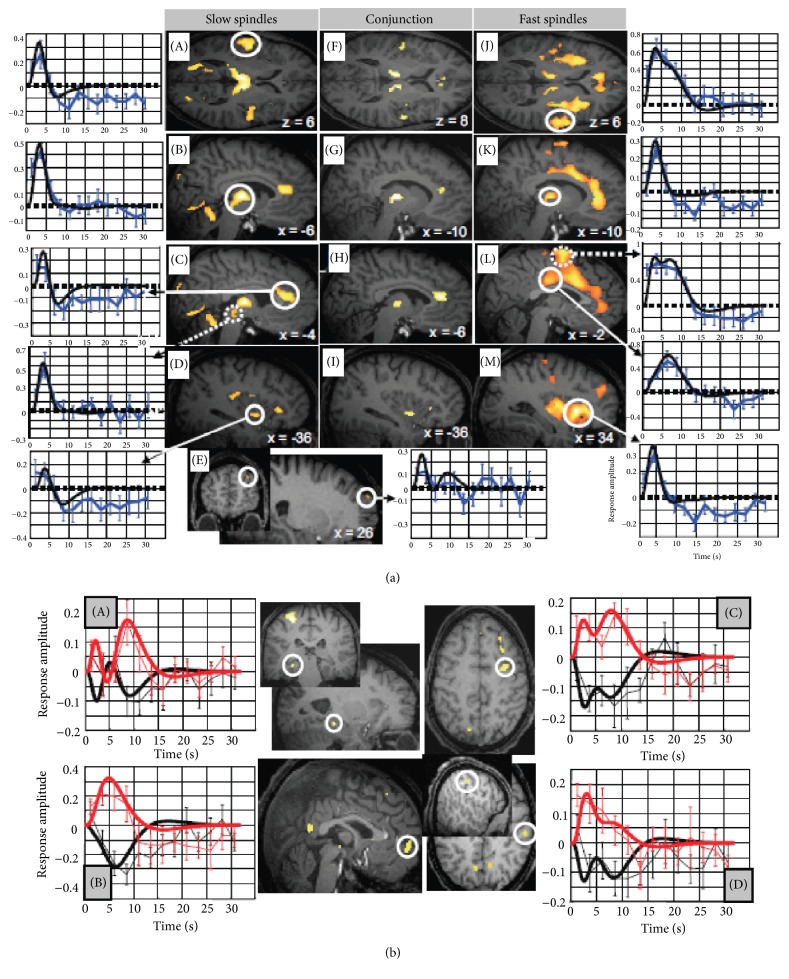
(a) Main effects of late slow and early fast spindles. (A–E left) fMRI responses to slow spindles displayed over an individual structural image normalized to the Montreal Neurological Institute space (*P* < 1). The leftmost panels show peristimulus time histograms (PSTHs) depicting the responses in auditory cortices (circled) (A), thalamus (B), anterior cingulate (circled) and midbrain tegmentum (dotted) (C), anterior insula (D), and superior frontal gyrus (E). The PSTH (solid blue line; blue error bars reflect the SEM) depicts the mean response across spindles of the corresponding voxel, irrespective of contrast based on a finite impulse response refit. The fitted response is drawn in black. (F–I center) Conjunction analysis of slow and fast sleep spindles. (J–M right) fMRI responses to fast spindles (*P*_uncorrected_ < 0.001). The right most panels show PSTHs depicting the response in superior temporal gyri (J), thalami (K), midcingulate cortex (circled) and SMA (dotted) (L), and anterior insula (M). (b) Differential fMRI activity between fast and slow spindles. Larger brain responses for fast (red) than slow (black) spindles were revealed in the hippocampus (A), mesial prefrontal cortex (B), precentral gyrus (C), and postcentral gyrus (D). Peristimulus time histograms show mean response of the corresponding voxels (dotted lines; error bars show SEM) and the corresponding fitted responses (continuous lines). Modified from [34].

From fMRI studies, a more general view of brain areas activated during sleep spindles also emerges [34, 71, 72], with the distinction of late slow frontal and early fast centroparietal spindles (Figure 8). The distribution of brain activity during both these spindle types have significant positive responses in the thalamus, cortex (paralimbic area, anterior cingulate cortex, and the left insula), and neocortex (bilateral response in the superior temporal gyrus) [34].

The activity of the early fast centroparietal spindles seems to be more constrained in the thalamus but extended at the cortical level (orbitofrontal and middle frontal areas, precentral and middle frontal gyri, SMA, and midcingulate cortex ventral to the cingulate motor zones) and in hippocampus [34, 71, 72]. During late slow frontal spindles, the brain activation resembles the common activity pattern with significant responses identified in the thalami, anterior cingulate, insular, and auditory cortices [34]. Compared to late slow frontal spindles (Figure 8(b)), the activations relative to early fast centroparietal spindles were significantly higher in orbital and middle frontal, precentral and postcentral, and insular cortices but also in mesial prefrontral cortex and hippocampus [34, 72].

The reasons for this complex topographic distribution are currently debated and include aspects of neocortical propagation and resonance, different contributions of thalamic nuclei and focal versus distributed thalamocortical projections from first- and higher-order thalamic nuclei, and the possibility of several spindle-generating loci. Most of these characteristics have also been observed from source reconstruction models of MEG and EEG recordings [38, 49, 73].

From this last section, we can conclude that at least two kinds of spindles exist: early fast centroparietal spindles and late slow frontal spindles. Both spindle types are distinguishable temporally [30, 38] and spectrally [20] as well as functionally since they are implied in different brain processing [15, 31] and respond differently to pharmacological manipulation [35]. Moreover, they seem to be initiated from different part in the brain despite a common origin in the thalamocortical loop [34, 49, 71, 72]. Finally, both spindle types would have a different dynamic over the scalp with early fast centroparietal spindles more locally synchronized and late slow frontal spindles more globally synchronized [38].

## 3. Automatic Spindle Detection Method

Traditionally, sleep experts proceed by visual inspection but this task is time consuming and prone to intra- and interscorer variability. It is also heavily dependent on the scorer's ability to visually distinguish spindles among varying EEG background activity. The need for an “automatic spindle detection method” (ASDM) is linked to the increase in interest for sleep spindles and the ever increasing amount of digital data to be analyzed. On the positive side an automated method allows for a detection which is faster, more reproducible, and systematic. However the issue with automated methods is their weak robustness when spindles characteristics change and the weak agreement with sleep experts [26].

**Figure 9:**
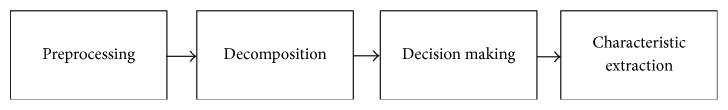
General workflow of an “automatic sleep spindle detection method” (ASDM). It is composed of 4 modules: preprocessing, decomposition, decision making, and characteristic extraction. The latter is optional.

The aim of this section is to describe and comment on automatic spindle detection approaches, through examples of published ASDM; ASDM are mainly characterized by their signal decomposition and decision making processes. Before these, a preprocessing step is usually necessary to prepare data. Finally, spindle characteristics can also be provided after the detection itself. A typical ASDM thus processes the EEG signal, channel by channel, via five serially connected modules, as shown in Figure 9.

From the spindle properties described in the previous sections, we can propose that a spindle should be detected as follows:

- a discrete burst of oscillations, that is, not a continuous activity;
- oscillations with frequency going from 9 to 16 Hz or any explicitly specified range within this span;
- a nonstationary wave due to a possible waxing and waning shape;
- local or synchronized event over the scalp: due to its focal apparition in deeper area, the spindle can also appear at scalp level as a superposition of several independent events.

The various ASDM described and cited in Sections 3.1 to 3.3 are summarized in a Supplementary Table S1 in Supplementary Material available online at http://dx.doi.org/10.1155/2016/6783812.

### 3.1. Preprocessing

In addition to bandpass filtering and signal downsampling [34, 74], preprocessing deals mainly with nonoscillatory transients. These activities can result from both measurements artifacts and nonrhythmic brain activities. By filtering data, these distortions are amplified making the interpretation of time-frequency analysis more complex and leading to spurious oscillatory activity [75, 76]. To address this issue, Parekh proposed the Dual-Basis Pursuit Denoising (Dual-BPD) method: it consists of nonlinearly decomposing the raw EEG signal into nonoscillatory transient and sustained rhythmic oscillation components using long and short windows for the STFT [77]. This technique was applied prior to filtering with 5 different methods [17, 78–81] and improved the spindle detection methods, increasing the number of truly detected events and reducing the number of falsely detected events [77]. Finally, specific artifacts and/or high alpha activity detection methods were developed [82–85] to clean up the signal or exclude noisy segments before spindle detection. This is why ASDM have been tested on specific artifact-free EEG periods [86, 87]. Therefore the preprocessing stage in a spindle detection method should ideally follow these successive steps:

1. a decomposition method such as the Dual-BPD to remove as many transients as possible;
2. bandpass filtering to remove slow fluctuations due to artifacts [88] and high frequencies to allow decimation without aliasing;
3. downsampling to reduce computing time;
4. artifacts and high alpha activity rejection.

### 3.2. Decomposition

The decomposition module consists in delimiting the events of interest in temporal and frequential domain before the feature extraction. It can either be operated directly on the preprocessed signal with spectral analysis and time-windowing (simple decomposition) or be preceded by some signal transformations (complex decomposition).

#### 3.2.1. Simple Decomposition

The order of operations (spectral analysis and time-windowing) determines if the features extracted are of temporal (temporal analysis) or spectral (spectral analysis) nature.

With the exception of waveform morphology for spindle detection (WMSP) [89], in temporal analysis, data are usually filtered first. This is typically done with a classical filter (e.g., Butterworth or Gauss filters). In this case, the bandpass filtering is applied in one frequency band [17, 63, 79, 83, 84, 86, 87, 90–93] or multiple ones, for example, to distinguish slow and fast spindles [34]. As frequencies also vary across individuals [20], filtering procedures, adapted to the specific frequency bands of spindles and individuals, have also been developed [80, 94]. Nevertheless spindle frequencies also slowly change overnight (Section 2.2) and filtering in narrow frequency bands can induce distortions in the signal. As an alternative to classical filtering techniques, a few approaches use wavelet filters [74, 95]. The Wavelet Transform (WT) is an efficient tool to decompose a signal into a fundamental set of components and obtain subband localization [96].

Prior to feature extraction, potential events still have to be temporally segmented, for example, with a moving time window. Then boundaries of potential events are delimited approximately by one (or more) segment(s) or determined with fixed or adaptive (e.g., percentile) thresholds applied on the envelope of the filtered signal. The envelope can be estimated either by the absolute value of the signal or the Teager Energy Operator (TEO) [97]. The TEO computes the instantaneous energy and is sensitive to changes in both frequency and noise.

With spectral analysis, the signal is first temporally windowed before being transformed into spectral data with Fast Fourier Transform (FFT) [82–84, 98–101]. The time window usually lasts 0.5 s, a result from a trade-off between spectral resolution and stationarity condition (≤0.5 s). When the time delimitation is done with fixed time windows, the spindle boundaries are estimated only approximately, despite the fact that “duration” is essential in spindle analysis (Section 3.4).

#### 3.2.2. Complex Decomposition

To limit frequential (resp., temporal) uncertainty in event delimitation due to fixed predefined frequency bands (resp., time-window length), some preliminary transformations can be used. These allow the simultaneous extraction of temporal and spectral components (time-frequency analysis) or the direct estimation of features from a model-based approach (model-based decomposition).

(*1) Time-Frequency Analysis*. Time-frequency analyses are computed from transformation derived from linear decomposition methods such as Short Time Fourier Transform (STFT) [74, 89, 102], Continuous Wavelet Transform (CWT) [78], and Matching Pursuit (MP) [36, 103, 104] or nonlinear decomposition methods such as Complex Demodulation (CD) [105], Hilbert Huang Transform (HHT) [106], and SynchroSqueezed Transform (SST) [85].

Both STFT and CWT have to deal with the Heisenberg principle (fix ratio between time and frequency resolution). The resolution of STFT is regulated by the length of the analysis windows and is maintained over all frequencies whereas the CWT use wavelet filters to obtain higher frequency resolution in lower frequency and higher temporal resolution in higher frequency. The main drawback of the CWT is that the ideal resolution depends on the a priori good choice of the wavelet filters (“mother wavelet”) [107]. To achieve better time-frequency resolution, iterative methods are used to either reconstruct the original signal from well-known functions (MP) or deconstruct the original signal in main components to be evaluated (HHT, SST, and CD).

The Matching Pursuit breaks down the analyzed signal into a weighted sum of known functions from a set of “atoms” called Dictionary [107]. Separating the oscillatory part from the transient one was shown to be an efficient approach, at least for noise-free (or artificial) signal, but it could be suboptimal in low SNR conditions [76]. This adaptive approach assumes that the signal components are well represented by atoms of the Dictionary but this is not necessarily the case in real data. Consequently, nuances in sleep spindles characteristics could induce distortions in successive iterations. Improvements of this method rely on creating a Dictionary whose functions are more adapted to EEG sleep patterns to induce less distortions in the iterative decomposition. For example, “chirplets” [36] have time-varying oscillations, that is, faster/slower waves over time. Another possibility is the use of a self-updating Dictionary whose content increases with each detection performed [108].

The Hilbert Huang Transform (HHT) decomposes the signal into intrinsic mode functions (IMF) via the Empirical Mode Decomposition (EMD) with good time resolution [109] and uses the Hilbert Transform to estimate instantaneous amplitude and frequency [110]. HHT is limited to narrowband signals and is very sensitive to noise [111]. To address this issue, some improvements have been proposed [112]. Another possibility is the Complex Demodulation (CD) approach that isolates in the spectral domain the signal of interest [113]. The main drawback of this method is the necessity to a priori specify the frequency band corresponding to the signal of interest [114].

The SynchroSqueezed Transform is an approach that can be associated with the EMD but is built differently. It is a special case of reallocation method that aims to sharpen a time-frequency representation according to local behaviors around nonzero activity [75].

**Figure 10:**
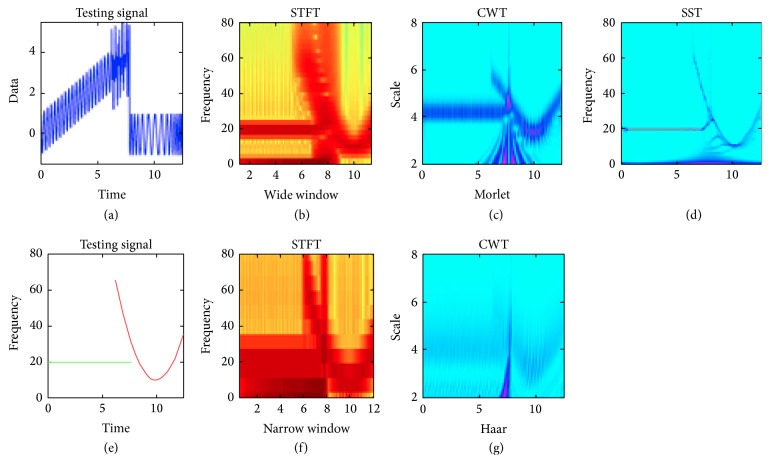
Time-frequency analysis. The testing signal (upper left corner) is composed of two kinds of oscillations (constant and variant) with a sharp transition in between. Its ideal spectral representation is shown on the lower left corner. Due to their limited time-frequency resolution, the sharp transition induces blurring in the time-frequency representation of STFT and CWT whatever the window size and the mother wavelet, respectively. On the contrary, the SST approach is still able to correctly follow the instantaneous variations in frequency. Modified from [75].

Huupponen et al. [115] compared some of these methods and showed that the MP method performed better than the STFT (with zero padding). Nevertheless, the MP was implemented with the same functions as the ones used to create the testing data (synthetic signal with known spindles characteristics) which could be biasing the assessment. A more recent study, also assessed with synthetic data and simulated spindles, showed that the HHT was better at detecting spindles than the MP and CD methods [116]. A last comparison was performed by Daubechies et al. [75] who show differences between STFT, CWT, and SST. In this case, the signal is composed of two kinds of oscillations (constant and variant) with a sharp transition in between (Figure 10). This induces blurring effect in the time-frequency representation of STFT and CWT because of their limited time-frequency resolution. On the contrary, the SST approach is still able to correctly follow the instantaneous variations in frequency.

To summarize, EEG signal contains transient activities inducing blurred representations in STFT and CWT analysis. These two approaches cannot clearly distinguish diffused “background” activities in the spindle frequency band from well-defined spindles [75]. MP has a concise description of the signal with a relatively small number of atoms but its adaptive iteration method and its limited Dictionary prevents nuances in spindles analysis. However, new methods could lift this limitation [108]. CD computes activities in well-defined frequency bands but needs a priori information about the carrier frequency [113, 114]. HHT is very sensitive to noise but numerous enhancements exist and good preprocessing such as the Dual-DBP (Section 3.1) could address this issue. Finally the SST method seems to be the most robust and precise method to discriminate transient activity.

(*2) Model-Based Decomposition*. The model-based decomposition uses mathematical models to transform the signal into a new set of components such as Principal Component Analysis [117] and linear models [118–121].

The Principal Component Analysis is a statistical procedure converting correlated variables into a set of uncorrelated variables decomposing the initial signal into its main components [122]. The ability to distinguish uncorrelated components is a very interesting advantage. Nevertheless, to be interpreted, these unlabeled components need more laborious decision making and/or preliminary analysis [117].

A linear model is a mathematical equation whose complexity depends on its order. For example, the autoregressive model of order *n*, denoted by AR(*n*), uses the *n* last values in a given time series to estimate the next value. The AR model is constrained to stationary signal and is unable to track the slow change in the spectrum [119]. To address this issue, the Adaptive-AR(*n*) estimates parameters of its model by using the Least Mean Square (LMS) method for each sample. A last model is the ARMA model made of an autoregressive and a moving average part of orders *p* and *q*, respectively, and noted ARMA(*p*, *q*). This model is more complex but could provide a better representation of the sleep spindle [123, 124]. The main drawback of linear modelizations is that the quality of data representation depends on the model order whose optimal values are only local.

### 3.3. Decision Making

Decision making relies on either thresholding approaches, with fixed or adaptive parameters, or multiparameters approaches combining different features simultaneously.

#### 3.3.1. Thresholding

Thresholds can be fixed empirically from limited training dataset (fixed threshold) [90, 103, 106, 120, 121] or self-adjusting via some data derived statistics (adaptive threshold) [17, 34, 79, 80, 83–87, 93–95, 104, 105]. To refine adaptive thresholds, some detectors must be informed by prior knowledge about sleep stages [63, 78–80, 94] or previously detected spindles [85, 87, 95]. ASDM with fixed thresholds are unable to account for all spindle variabilities. Depending on the subject, these detectors show weak sensitivity or specificity and are therefore unreliable. Importantly one should not use a fixed threshold on specific characteristics to detect spindles and then produce any statistics based on the same characteristics. In other words, this circular reasoning implies that you can only find what you are looking for.

Adaptive thresholds are computed either once for all from a set of a priori data (e.g., NREM sleep periods or sleep spindles visually detected) or specifically for each putative spindle from a determined time window centered on it, for example, to compare spindle activity to its background. If well defined, the former approach can be sufficient to correctly detect spindles for sleep classification (Section 3.4). However, overnight changes of spindle characteristics are better characterized with the latter. Methods relying on prior data usually only consider light sleep (N2) or sleep spindles detected in N2 for the definition of spindle detection parameters. Such approach would thus be constrained and biased to the a priori set of spindles considered and could introduce subjectivity if the spindle set was defined manually. This approach is thus suboptimal for the other sleep stages, as spindle characteristics vary across sleep stages (Section 2.2).

To avoid such biased or suboptimal detection, a few ASDM propose the use of multiple features combined with more complex decision-making (Section 3.3.2). These features are directly taken from time series [92] or spectral data [101, 102] or need preliminary transformations such as linear model parameters [118–121] or principal components derived from PCA [117].

#### 3.3.2. Multiparameters Approaches

Machine learning techniques operate by building a model from example inputs in order to make data-driven predictions or decisions. Machine learning can be described as supervised or unsupervised depending on the nature of the learning process used. Supervised learning uses a training dataset composed of examples of labeled data, that is, with data (input) and their associated label (output). The model then learns the mapping from input to output from these examples. On the contrary with unsupervised learning algorithm, no labels are provided and the model has to learn the structure of the input data on its own.

A very simple supervised learning method used by some ASDM is the decision tree which can combine different detection methods with adapted and/or fixed thresholds [74, 89]. Fuzzy logic is a form of probabilistic decision tree that induces the certainty with which the spindles are detected [91, 98, 106].

More complex supervised learning methods are also used in many ASDM, for example, the “Multilayer Perceptron” (MLP) [92, 102, 118, 119] and the “Discrete Perceptron” (DP) [119] methods. These feedforward artificial neural network models consist of multiple layers of nodes in a directed graph with each layer fully connected to the next one. Such methods require a good balance between complexity (numbers of neurons and layers) and computation time. The “Support Vector Machine” (SVM) [101, 102, 118, 119] performs linear or nonlinear classification depending on the kernel function used [125]. The performance of SVM and MLP is difficult to compare because of MLP's parametrization. However, SVM is considered more robust than MLP [102, 118, 119].

Statistical models tend to represent the data generation process of a system, like the Bayesian model [82, 98, 117] or “Hidden Markov Model” (HMM) [101], and can be used for event recognition. Bayesian models are able to represent induced dependencies between variables in the system whereas HMM point to cyclic dependencies. In these models, variables are linked via probabilistic distributions initially defined from a training dataset. We can also use such probabilistic distributions to estimate if the characteristics of a putative spindle are truly those of a spindle or not [84, 101].

The main weakness of supervised machine learning for spindle detection remains the training set. For a reliable and generalizable detection, the training dataset should include a sufficient number of representative spindles, that is, with a wide range of characteristics from young and elderly populations, healthy subjects, and patients (with neurological/psychiatric disorders), with and without experimental conditions (e.g., sleep deprived), and so forth [126]. The creation of such (training) dataset is itself an enormous and difficult task.

Unsupervised learning methods do not rely on prior data for training and aim at inferring a function to describe hidden structure from unlabeled data but were barely developed for spindle detection (e.g., neuronal gas and merge neuronal gas [100]). However, unsupervised learning would probably be the most robust method if features are well defined.

### 3.4. Features Extraction

It is important to understand which characteristics are essential in sleep spindle analysis. Indeed, we think that a distinction should be made between spindle properties and detection criteria to avoid the standardization of spindles detected and, consequently, bias further spindle analysis.

One usually extracts only useful characteristics depending on the aim of the analysis. As an example, to score sleep stages, no specific spindle characteristic is needed. In order to compare results from several sleep analyses, one should rely on exactly the same spindle characteristics.

#### 3.4.1. Individual Properties

Many sleep spindles analyses exploit individual properties to show differences between populations. For example, studies showed that the intraspindle frequency increases with age while spindle duration decreases [16, 17]. In total, we list four categories of individual properties: frequential, temporal, spatial, and dynamic (Table 1).

The “main frequency” of a spindle, that is, the average of instantaneous frequencies, can be used as unique value to characterize the spindle as slow or fast. This distinction is important as late slow and early fast spindles have specific functions in some brain processes (e.g., memory process [31]). “Intraspindle frequency” change can be expressed by its pace (Hz/s) and is either positive or negative for increasing or decreasing oscillation rate, respectively.

Spindle “duration,” defined as the elapsed time between the start and end point of the spindle, depends strongly on the decomposition method used in ASDM (Section 3.2) and is often poorly estimated compared to human [26]. This property, with the “intraspindle frequency,” is probably the most representative parameters of the thalamocortical process [126]. The “shape,” which is related to cortical process [126], can be described by two measures: skewness and kurtosis. The skewness is positive (resp., negative) when the maximal amplitude of sleep spindle is shifted to the left (resp., right) whereas kurtosis value is positive (resp., negative) for spindles with sharper (resp., flatter) envelope than a normal distribution.

The spatial aspect of a sleep spindle needs multiple channels analysis for “scalp localization” and an inversion model for “source localization.”

Regarding the spindle dynamic, the “interplay” relates to the link between sleep spindles and SWO. This relation can be quantified as the duration between time points corresponding to maximum spindle peak and maximum negative SWO peak, the latter being taken as reference time (*t* = 0) [31] (Section 2.2.4). Positive (resp., negative) value would be given to “interplay” when the maximal amplitude of the sleep spindle precedes (resp., follows) this reference time. The “interplay” parameter is particularly interesting for any studies related to learning and memory processes [15] or spindles generation [5].

#### 3.4.2. Ensemble Properties

From all spindles detected (overnight) or part of them (e.g., during one NREM cycle), the following ensemble properties could be computed: the “count” is the number of spindles detected, the “density” is the number of spindles over time intervals (e.g., sleep stages and cycles), and the “rate” is the time between successive spindles. These properties are exploited in numerous analyses. For example, changes in “count” are related to sleep perturbation [127, 128], psychiatric disorders such as schizophrenia [129], and aging [16, 17]. Estimated over successive short 20 s epoch, the “density” brings out the fact that the number of sleep spindles is reduced after a sleep deprivation (confirming an inverse homeostatic relationship of sleep spindles and slow waves [130]). Finally, the “rate” is, for example, a characteristic which becomes more variable over ages [16].

#### 3.4.3. Detection Criteria

Most of the comparisons between human and automatic detection point to an overestimated number of automatically detected spindles [26, 131], suggesting a higher sensitivity of automatic detection compared to visual scoring. Though different mismatches (as compared to the same human consensus) are observed for each ASDM, depending on the approach, each ASDM is likely to confound spindle events with other specific types of EEG activity [26].

As described through Section 3, to determine if a bit of signal is a spindle or not, various features can be used and each ASDM combines them in different ways. Finding the optimal approach is still an open question; nevertheless some features are open to criticism. Most ASDM rely directly on sleep spindles properties for their detection and thus necessarily standardize sleep spindles detected. This introduces bias in further analysis and has big influences on performance. For example, the “shape” [85, 89, 106] is a severe criterion which probably decreases the sensitivity for the spindle detection. On the contrary, both “frequency” [74, 84] and “amplitude” criteria lead to more sensitivity detection but cannot distinguish sleep spindles from continuous sigma activity. To address this issue, some methods take into account the contextual aspect of the sleep spindles by using the power spectrum in specific frequency bands [82, 95, 98–100, 106]. Critically detection criteria based directly on the amplitude or its power spectrum should be avoided. Indeed their absolute value strongly depends on the recording setup as well as on the source localization and orientation. However the two most common detection criteria in ASDM are actually the amplitude [74, 78–80, 82, 83, 86, 87, 90–95, 103, 105, 106] and the root mean squared values (RMS) of power spectrum or filtered signal [17, 34, 63, 84, 104]. This may at least partly explain the lack of generalizability of ASDM.

Anecdotally the one criterion shared by all ASDM is the duration of the sleep spindle event. Although important, this aspect is often overlooked [26], despite its impact on the estimation of the other spindle features.

## 4. Assessment of the ASDM

The performance of a machine depends on the trade-off between its sensitivity (ability to detect all/most events of interest) and its specificity (ability to detect only/mostly events of interest). For example, when sleep spindle detection is used for sleep staging classification, sleep spindle detection should be more specific than sensitive in order to detect spindles (at least enough of them) in stage 2 and avoid falsely detected events in the other stages. However, when the primary goal is the characterization of the spindle activity, for example, to measure the effect of pharmacotherapy or neurological/psychiatric disorders, the sensitivity of spindle detection has to be increased, usually to the detriment of specificity [126].

In any case, in order to properly assess the performance of an ASDM, we need to compare it to some references, typically annotated databases, and rely on robust assessment statistics.

### 4.1. Gold Standard for Evaluation

Human raters are subjective and less sensitive but more specific compared to machines. In order to obtain a reference score which is both sensitive and specific, a consensus between different raters has to be generated: the “gold standard.” There exist different ways to generate such “gold standard” [82, 119, 132] and to account for the variability of human raters, Warby et al. [26] proposed the following:

(i) Human raters have to detect spindles and give to each detected event a confidence score (1 = definitively, 0.75 = probably, 0.5 = maybe/guessing, and 0 = no spindle).
(ii) For each sample, the mean of weighted confidence scores is computed.
(iii) A sample is considered as a spindle if the mean value is larger than some threshold (a threshold value of 0.25 was empirically found as being the most representative by [26]).

To assess the robustness of an ASDM a large database composed of heterogeneous populations (healthy versus pathologic, young versus elderly) [126] is required. To obtain such database, crowd-sourcing (examples of crowd-sourcing: the Montreal Archive of Sleep Studies (MASS): http://www.ceams-carsm.ca/en/MASS/ [133]; Physionet: https://www.physionet.org/; and DREAMS: http://www.tcts.fpms.ac.be/~devuyst/Databases/DatabaseSpindles/) is an efficient and fast way to amass data and raters and to benchmark many ASDM simultaneously [133–135]. Furthermore, freely available databases can be continuously scored or commented on by experts and nonexperts.

It is also crucial that ASDM implementations are available for others to use [26]. At least for scientist applications such an open-source software approach had several benefits: no time is wasted reimplementing a published method, results are then more easily reproduced, code reviewing will spot and correct bugs over time, and the method can be improved and new features added. Moreover, different methods could directly be compared on the same databases with the same assessment method [134].

### 4.2. Assessment Statistics

For a fair assessment of any ASDM, the data on which the method is tested should not have been used to set up the method itself. Training and testing a method on the same dataset is a clear case of “double dipping,” which leads to overestimated positive results but provides no information on the generalizability of the method to other datasets.

To assess the detection provided by the ASDM, it should be directly comparable with the gold standard and have the same temporal granularity, for example, 1 s time window. Typically, for each signal, the value “1” is attributed to all segments where the presence of a spindle is considered, “0” otherwise. Finally, both signals are compared and the following occurrences are counted: (i) true positives (“TP”); that is, the gold standard and the ASDM give “1”; (ii) true negatives (“TN”); that is, the gold standard and the ASDM give “0”; (iii) false positives (“FP”); that is, the gold standard detection is “0” whereas the ASDM gives “1”; (iv) false negatives (“FN”); that is, the gold standard detection is “1” whereas the ASDM gives “0.”

Over the course of a sleep EEG recording, there are many more time bins without spindle than with a spindle. Therefore standard matching measures such as specificity are not useful. For this reason, it is more appropriated to use the “Recall” and “Precision” parameters (1)-(2):

(1) Recall=TPTP+FN

(2) Precision=TPTP+FP.

“Recall” is the ratio between the number of spindle events correctly detected and the total number of spindle events considered by the gold standard. “Precision” is the ratio between the number of spindle events correctly detected and the total number of events detected by the machine. They are equivalent to the sensitivity and the positive predictive value (PPV), respectively. From these two measures, a single value can be derived the *F*1-score which is the harmonic mean of Precision and Recall:

(3) $$\begin{array}{c} F1=\frac{2*\text{Precision}*\text{Recall}}{\text{Precision}+\text{Recall}}. \end{array}$$

If *F*1 = 1, then the detection was 100% accurate with no false positive or false negative detected event. In a more realistic case, for example, Recall of 0.8 (corresponding to 25% of false negative detected event) and Precision of 0.9 (corresponding to 11% of false positive detected event) lead to *F*1 value of 0.85. To ease future comparison across literature, three statistic parameters (1)–(3) should be employed.

For a more complete detector evaluation, a second unit of comparisons is proposed: each individual spindle is considered as an event. To decide when 2 raters agreed on a common event, that is, spindle and detected event, a minimum of overlap between the spindles has to be fixed a priori. For a detected event to be considered as a match, the minimum overlap between the gold standard and detected spindle can, for example, be fixed at 20% [26]. The three statistics (1)–(3) defined above can be reexpressed in terms of events instead of 1 s time windows.

## 5. Discussion and Concluding Remarks

This review tried to define the sleep spindle as a simple pattern or dynamic event. The main brain processes implied in its generation have been described in order to define its physical parameters. Likewise, its topographic and dynamic aspects have been presented in order to understand how this phenomenon appears in the brain.

From this general description, a sleep spindle can be seen as an oscillatory mode that occurs transiently but the characteristics of this pattern are not strictly defined. A sleep spindle is regulated by slow oscillations and is itself the regulator of faster oscillations; otherwise, it belongs to a hierarchical nesting of oscillatory modes [61]. The sleep spindle is characterized by internal frequency changes. However, it is usually characterized by a unique frequency corresponding to the frequency with the maximal power. From its mean frequency, the sleep spindle has been classified as late slow frontal or early fast centroparietal. However, this differentiation is not so clear-cut in some individuals [20].

Sleep spindles are patterns of activity of great interest and automatic detection methods are essential for reproducible analysis. Indeed to better understand their functioning, their detection should be done in a systematic and robust way. By this way, comparisons between different populations (e.g., elderly versus young, health versus psychiatric/neurodegenerative disorders) will be more reliable.

In order to find an optimal method, this review described some ASDM and proposed an evaluation process. In the ASDM description, advantages and disadvantages of the main processes are listed. An important point is to delimit the event as precisely as possible to extract the characteristic features of the putative spindle; which ones are most specific is still unknown. Regarding decision making, machine learning and statistical models are more robust methods in the face of the large variability of spindles.

In conclusion the objective comparison of all the existing and future methods would require that their implementation (executable or source code) is made available and that we have access to databases with a large variety of marked recordings.

## Supplementary Material

The supplementary Material lists the automatic and semi-automatic sleep spindles detection methods described along the review. These methods are described by their main processing steps: ‘Decomposition', ‘Features' and Decision-making'.

## Figures and Tables

**Table 1:** Main characteristics of sleep spindle (SS). SWO: Slow Wave Oscillations; coord: coordinates.

Parameters of SS	Measurement	Units
* Individual properties *		

Main frequency	Mean intraspindle frequency	Hz
Intraspindle frequency	Rate of frequency changes	Hz/s
Duration	Time between start and end	s
Shape	Skewness and kurtosis	—
Scalp localization	EEG channel	coord(*x*, *y*)
Source localization	Spindle origin	coord(*x*, *y*, *z*)
Interplay	Delay between the nearest SWO and SS	s

* Ensemble properties *		

Count	Number of SS	—
Density	Count by min (within cycles and sleep stages)	min^−1^
Rate	Time interval between successive SS	s
