# Supplementary material for: Sleep Spindles as an Electrographic Element: Description and Automatic Detection Methods
Source: Neural Plast. 2016 Jul 11;2016:6783812. doi: 10.1155/2016/6783812 (PMC4958487; doi:10.1155/2016/6783812)
Supplement: Supplementary file 1 — The supplementary Material lists the automatic and semi-automatic sleep spindles detection methods described along the review. These methods are described by their main processing steps: ‘Decomposition', ‘Features' and Decision-making'. [file 6783812.f1.pdf]

Table S1: Main characteristics of Automatic Spindles Detection Methods (ASDM). When several decision makings or features are tested, the best one is underlined. Abbreviations: A_f: EEG signal filtered in the frequency band 'f', S_f: spectrum in the frequency band 'f', RMS: Root Mean Square, MA: Moving Average, AT: Adapted Threshold, FT: Fixed Threshold, CWT: Continous Wavelet Transform, WT: Wavelet Transform, FFT: Fast Fourier Transform, SST:SynchroSqueezed Transform, CD: Complex demodulation, CUSUM: Cumulative sum, PCA: Principal Component Analysis, ACF: Autocorrelation Function, (A)AR(n): n-order (Adaptative) Autoregressive model, MLE: Maximum Likelihood Estimation, AHC: Ascending Hierarchical Classification

**Decomposition**	**Features**	**Decision-making**
	**Schimicek, 1994 [90]**	
filtering	envelope(A_σ)	FT
	**Huupponen, 1999 [98]**	
segmentation<br>FFT	$R_\theta \propto \frac{S_\sigma}{S_\theta}$<br>$R_\alpha \propto \frac{S_\sigma}{S_\alpha}$<br>$R_\beta \propto \frac{S_\sigma}{S_\beta}$	fuzzy logic
	**Huupponen, 2000 [82]**	
SS-like		
filtering	peak(A_σ)	fuzzy logic $\Rightarrow$ SS-like
segmentation<br>FFT	artifact index<br>sleep index	
SS detection		
filtering	peak(A_σ)	AT from SS-like
	**Molle, 2002 [63]**	
filtering<br>segmentation	RMS(A_σ)	AT
	**Görür, 2002 [102]**	
STFT(2-64Hz)	32 STFT values	MLP or <u>SVM</u>
	**Görür, 2003 [118]**	
AR(16)	17 AR parameters	MLP or <u>SVM</u>
	**Olbrich, 2004 [120]**	
AR(8)<br>decomposition [136]	oscillatory modes<br>damping factors	FT
	**Held, 2004 [91]**	

Table S1: Main characteristics of ASDM

**Decomposition**	**Features**	**Decision-making**
filtering	peak(A_σ)	fuzzy logic ⇒ Out1
filtering (<17Hz)	amplitude, frequency, symmetry	FT ⇒ Out2
	Out 1-2	Out1 & Out2
**Acir, 2004 [119]**		
AAR(15)	16 AAR parameters	DP + (SVM or MLP)
**Clemens, 2005 [86]**		
filtering	envelope(A_σ)	AT
**Ventouras, 2005 [92]**		
SS-like		
filtering	A_σ	MLP ⇒ SS-Like
SS detection		
sA_σ = MA	peak(sA_σ)	FT
**Bodizs, 2005 [94]**		
2 adapted filtering	envelope($A_{\sigma_{slow}}$)<br>envelope($A_{\sigma_{fast}}$)	AT from NREM
**Schonwald, 2006 [103]**		
MP	MP atoms	FT
**Devuyst, 2006 [83]**		
filtering	envelope(A_σ)	AT
**Huupponen, 2007 [99]: comparison of 4 methods**		
detector 1		
segmentation<br>FFT	$\Sigma\text{I} \propto \frac{(S_\sigma)}{S_\sigma+S_\beta}$	fuzzy logic ⇒ SS-like
filtering	ΣI, A_σ	AT from SS-like
detector 2		
FFT	ΣI	FT
detector 3 [82]		
filtering	A_σ	AT
detector 4		
filtering	A_σ	FT

Table S1: Main characteristics of ASDM

Decomposition	Features	Decision-making
	**Èstevez, 2007 [100]**	
segmentation<br>FFT	$S_{\delta-\theta}$, S_α, S_σ, $S_{\sigma-\beta1}$,$S_{\beta2}$	NG or <u>MNG</u>
	**Ferrarelli, 2007 [93]**	
filtering	envelope(A_σ)	AT
	**Schabus, 2007 [34]**	
filtering	RMS($A_{\sigma_{slow}}$),<br>RMS($A_{\sigma_{fast}}$)	AT
	**Bodizs, 2009 [80]**	
adapted filtering	envelope (A_{slow})<br>envelope (A_{fast})	AT
	**Ahmed, 2009 [95]**	
WT	$\frac{S_\alpha}{S_\delta}$	AT ⇒ Out1
TEO + ACF	envelope	AT ⇒ Out2
	Out 1-2	Out1 & Out2
	**Duman, 2009 [74]**	
localization		
STFT	frequency	FT ⇒ Out1
sub-localization		
A_σ from WT		
MUSIC(A_σ)	frequency	FT ⇒ Out2
TEO(A_σ)	amplitude	AT ⇒ Out3
detection		
	Out 1-3	AND(Out1,Out2,Out3)
	**Causa, 2010 [106]**	
SS-Zones(30s)		
FFT<br>Average Power (AP)	AP_δ,AP_σ,$AP_{\beta2}$	FT
SS candidates (events)		
HHT	amplitude, frequency	fuzzy logic

Table S1: Main characteristics of ASDM

Decomposition	Features	Decision-making
SS-like		
peak detection	amplitude, frequency, symmetry	AT
detection		
	AP_δ,AP_σ	decision tree
**Ray, 2010 [87]**		
filtering	peak (A_σ)	AT
**da Costa 2012 (WSMD) [137]**		
peak detection	frequency, shape	FT
**da Costa 2012 [89]**		
detector 1		
STFT	S^2	AT $\Rightarrow$ Out1
CWT	$\|S_\sigma\|$	AT $\Rightarrow$ Out2
peak detection	frequency, shape	FT $\Rightarrow$ Out3
	Out 1-3	Out1 & Out2 & Out3
**Wamsley, 2012 [78]**		
CWT	MA(S_σ)	AT
**Wendt, 2012 [79]**		
filtering	envelope(A_σ)	AT
**Babadi, 2012 [117]**		
Instantaneous Probabilities (IP)		
PCA	expansion coefficients	Bayesian inferences $\Rightarrow$ IP
SS detection		
MA(IP)	smoothed(IP)	FT
**Mporas, 2013: [101] fusion of 2 detectors**		
FFT	11 S_σ + 11 $\frac{\partial(S_\sigma)}{\partial(t)}$	SVM, HMM
**Martin, 2013 [17]**		
filtering	RMS(A_σ)	AT
**Nonclercq, 2013 [84]**		
pre-selection		
FFT	amplitude, frequency	FT and AT

Table S1: Main characteristics of ASDM

**Decomposition**	**Features**	**Decision-making**
detection		
modelization (MLE)	amplitude, frequency	AT
**Warby, 2014 [26]: Comparison of [17, 63, 78–80, 93]**		
**O'Reilly, 2015 [138]: multichannel analysis**		
sensitivity		
average over channels<br>CWT<br>wavelet ridges<br>peak detection	S,<br>$\Sigma I \propto \frac{S_\sigma}{S_\theta + S_{\beta 1}}$	AT
specificity		
PCA	localization<br>$\mathrm{F} = \frac{freq-10}{range_\sigma}$	AHC + AT
**Durka, 2015 [104]**		
adapted MP	RMS(S_σ)	AT
**Ray, 2015 [105]**		
CD	Z-score(S_σ)	AT
**Kabir, 2015 [85]**		
SS-Like		
SST<br>CUSUM<br>zero-crossings	duration	FT ⇒ SS-like
detection		
peak detection	regularity index	FT
